# Potential TSPO Ligand and Photooxidation Quencher Isorenieratene from Arctic Ocean *Rhodococcus* sp. B7740

**DOI:** 10.3390/md17060316

**Published:** 2019-05-29

**Authors:** Yashu Chen, Mengyao Guo, Jifang Yang, Jigang Chen, Bijun Xie, Zhida Sun

**Affiliations:** 1 Natural Product Laboratory, Department of Food Science and Technology, Huazhong Agricultural University, Wuhan 430070, China; yashuchen@sina.com (Y.C.); bijunxie@sina.com (B.X.); 2 Agricultural Bioinformatics Key Laboratory of Hubei Province, College of informatics, Huazhong Agricultural University, Wuhan 430070, China; guomengyao96@gmail.com; 3 College of Biological and Environmental Science, Zhejiang Wanli University, Ningbo 315100, China; jfkwlq@163.com (J.Y.); genomic@163.com (J.C.)

**Keywords:** isorenieratene, macular xanthophylls, TSPO, EPR, antioxidant and anti-UVB radiation

## Abstract

Due to its special aromatic structure, isorenieratene is thought to be an active natural antioxidant and photo/UV damage inhibitor. In this work, isorenieratene that was extracted from Rhodococcus sp. B7740 isolated from the Arctic Ocean, showed excellent scavenging ability of both singlet oxygen and hydroxyl radical in the UVB-induced auto-oxidation process using the EPR method. Within an ARPE-19 cell model damaged by UVB radiation, isorenieratene showed fine protective effects (1.13 ± 0.03 fold) compared with macular xanthophylls (MXs) through upregulating of *tspo*. The molecular docking was firstly performed to investigate the interaction of isorenieratene with TSPO as a special ligand. Results showed isorenieratene might form a better binding conformation (S-score −8.5438) than MXs and indicate that isorenieratene not only can function as a direct antioxidant but also activate *tspo* in ARPE-19 cells. Thus, isorenieratene might ease the UV-related damages including age-related macular degeneration (AMD).

## 1. Introduction

Age-related macular degeneration (AMD) holds serious health threats to the elderly population and has a substantial chance leading to permanent loss of vision [1]. However, there are no effective methods and measures to control or reverse the occurrence of the disease, let alone specific therapeutic drugs. It is, therefore, important to find ways to slow down the pathological changes to delay the progression of the disease [2]. Although there have been many studies on the pathogenesis and treatment of AMD, its pathogenesis is still unclear. However it is mostly believed to be related with pathological issues like oxidative stress, inflammation and mitochondria dysfunction [3].

**Figure 1:**
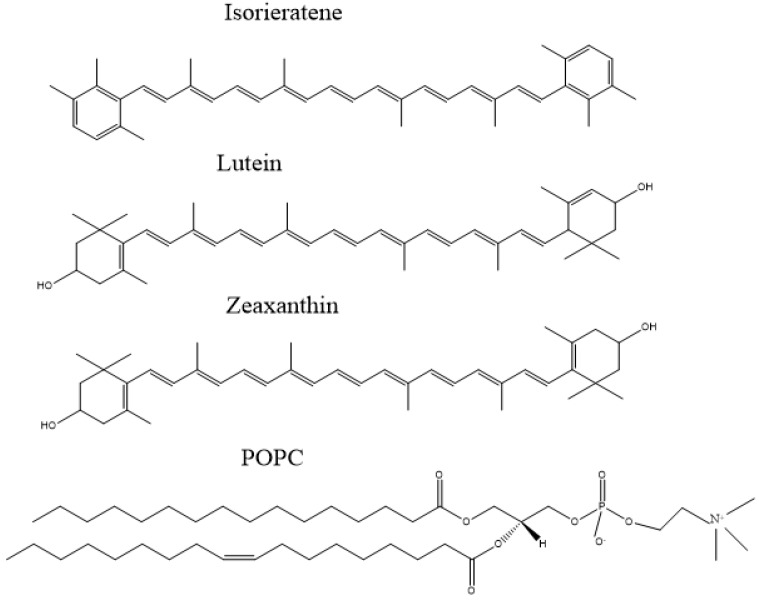
molecule structure of isorenieratene, lutein, zeaxanthin and POPC, respectively.

Ultraviolet (UV) radiation is a risk factor for most kinds of skin and ocular diseases including skin cancer, cataract and AMD. As a part of the light spectrum, UV radiation has a wavelength approximately between 200 and 400 nm. Although the cornea and lens absorb most UVA (320–400nm) and UVB (280–320nm), there is still a small fraction, particularly UVB, reaching the retina. Both the direct irradiation damaging effects to eye tissues and the UV-dependent secondary reactions make UV a main trigger for AMD [4]. Additionally, oxidative stress remained one of the most destructive secondary reactions of UV radiation. The formed reactive oxygen species (ROS) including singlet oxygen and free radicals, are easily accumulated in polyunsaturated phospholipids within a membrane location in retina [5]. Notably, macular xanthophylls (MXs), lutein and zeaxanthin (Figure 1) etc., are also selectively located in the same most vulnerable domains, susceptible to chemical bleaching and act as lipid antioxidants [5,6]. 

Lots of studies confirmed the impact of high consumption of carotenoids on the lower risks of AMD in the elderly. Carotenoid is one kind of natural essential pigment, which exists in almost all complex or unicellular organisms. For example, MXs also can be produced and collected from marine origin algal and microorganism [7,8,9]. They are presented in human diets as a nutrient enhancer, colorful agent, even medicine [10]. One of the medical applications of carotenoids is their important role in protecting the eye function. For example, MXs are selectively accumulated in retina to alleviate light damage. In addition to MXs, researches also demonstrated the preventing effects of many other natural pigments on AMD, such as β-carotene, astaxanthin, and anthocyanin [11,12,13]. However, one special carotenoid exists in the human diet, isorenieratene (Figure 1), whose potential effects on AMD has never been studied. 

According to our recent work, Rhodococcus sp. B7740 from the Arctic Ocean can produce amounts of menaquinones and special aromatic carotenoids including isorenieratene [10,14]. As hydrocarbon carotenoid, unlike β-carotene with two β rings, isorenieratene has special aromatic structure (two ϕ rings) thus might possess different bio-active functions. Reported data showed that isorenieratene possessed higher stability against oxidation stress than common plant source carotenoids [10]. In addition, benzene rings could increase its ability of UV resistance [15]. This provides the foundation for the conception and design of this article. Moreover, the wide research niche of this special marine origin carotenoid made us focus on its potential biologic functions and internal mechanisms. 

To investigate the potential effect of isorenieratene in the vulnerable polyunsaturated phospholipid domain, the multilamellar vesicles model using 1-palmitoyl-2-oleoylphosphatidylcholine (POPC, Figure 1), was applied in our research under UVB radiation [16]. Meanwhile, electron paramagnetic resonance (EPR) analysis, a sharp method to monitor free radicals, was performed to evaluate the scavenging ROS effects of isorenieratene compared with MXs.

In the mean time, the retinal-pigmented epithelial (RPE) layer, a major ocular tissue, is known to play an important role in the etiology of AMD. Cultured RPE cells exposure to UVB showed multiple cellular pathological features such as the reduced cell viability, increased cell apoptosis, reactive oxygen species (ROS) accumulation and mitochondrial dysfunction [4,17,18]. Thus, in this study, the defense effects of isorenieratene against UV-induced damage upon the ARPE-19 cell model was applied and also compared with MXs. It’s noteworthy that TSPO, an 18 kDa translocator protein, is located on the mitochondrial membrane in the cell. Research showed that TSPO-/-RPE cells possessed a significantly higher amount of ROS, lower cell viabilities and higher cholesterol efflux [19]. Thus ligands, which could bind with this specific protein, might contribute to the prevention of AMD. Additionally, stimulating over-expression of *tspo* in RPE cells by special ligands could be a new pharmacological way to treat early AMD patients. Mages et al. found that the special TSPO ligand XBD173 could protect inner retinal neurons by attenuating the glial response [20]. However, there is no research talking about the possible effects of MXs or other natural drugs as specific TSPO ligand in the ARPE-19 cell. Based on that, the potential effects of isorenieratene and MXs on ARPE-19 cells in the perspective of *tspo* expression were explored. Their special interacting mechanisms as TSPO ligand, were also studied and compared for the first time. Combined results clearly demonstrated the outstanding function of isorenieratene in eye protection. The aim of this study is to explore the potential bio-activity of this special aromatic carotenoid from the Arctic Ocean. We presume this aromatic carotenoid would have a promising application in the field of medicine.

## 2. Results and Discussion

### 2.1. EPR Analysis 

#### 2.1.1. Singlet Oxygen Measurement

**Figure 2:**
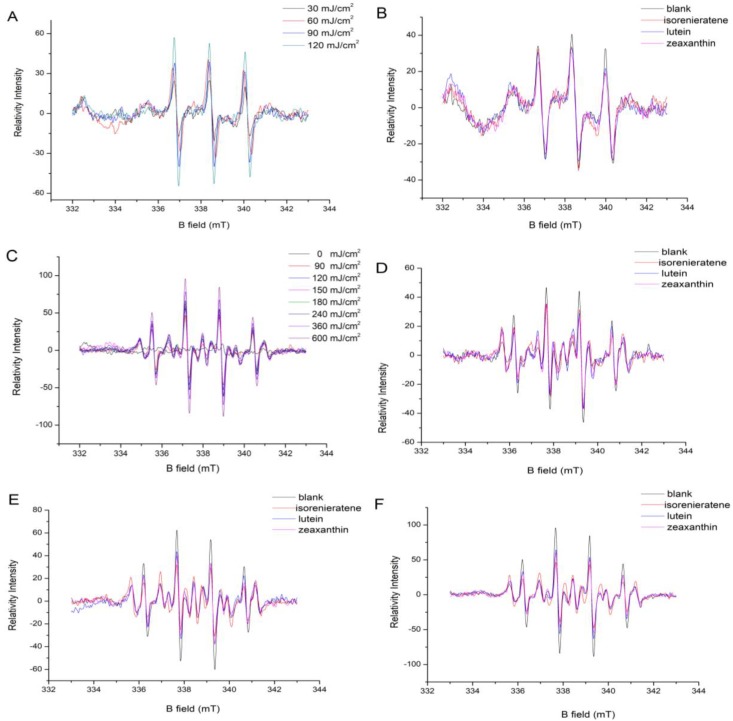
The singlet oxygen accumulations in model liposomes after different doses of UVB irradiation; (**A**) the inhibiting effects of three different kinds of carotenoids on the accumulation of singlet oxygen after 60 (**B**) mJ/cm2 UVB radiations. The hydroxyl radicals accumulations in model liposomes after different doses of UVB irradiation; (**C**) the inhibiting effects of three different kinds of carotenoids on the accumulation of hydroxyl radicals after 90 (**D**), 180 (**E**) and 600 (**F**) mJ/cm2 UVB radiations, respectively.

After increased UVB irradiation, the singlet oxygen accumulation trend was presented in Figure 2A. Carotenoids were reported to play an important role in quenching singlet oxygen in retina. Figure S1A and Figure 2B showed the inhibiting effects of three different kinds of carotenoids on the accumulation of singlet oxygen after 30 and 60 mJ/cm^2^ UVB radiations, respectively. These two figures show that isorenieratene possesses the similar quenching effect with macular xanthophylls (lutein and zeaxanthin). When the UVB radiation dose increased to 90 mJ/cm^2^, all three kinds of carotenoids showed no inhibitive effects on single oxygen accumulation (Figure S1B).

#### 2.1.2. Hydroxyl Radical Measurement

Researchers have long focused on the protection effect of carotenoids on the photo-induced retina damage [16]. The protective role of carotenoids may be related to antioxidant effects in retina membranes, involving both scavenging of free radicals and quenching of singlet oxygen. Figure 2C showed the increase trend of hydroxyl radicals in model liposomes system under increased dose of UVB radiations (90, 120, 150, 180, 240, 360 and 600 mJ/cm^2^, respectively). The addition of carotenoids was monitored to explore its effect on free radical formation in the photooxidation process. Figure 2D showed the EPR spectrum of hydroxyl radicals in model liposomes system added with three different carotenoids under 90 mJ/cm^2^ UVB radiations. The result showed that three kinds of carotenoids posed evident threat to liposomes photooxidation with reduced formation of hydroxyl radical under UVB radiation. Isorenieratene showed no difference in the inhibitive effect compared with lutein and zeaxanthin under 90 mJ/cm^2^ UVB radiations. Figure 2E,F showed the inhibition effects of three different kinds of carotenoids in the model liposomes system after 180 and 600 mJ/cm^2^ UVB radiations, respectively. Both figures showed that with the increased UVB radiation dose, isorenieraten exhibited better inhibitive effect against hydroxyl radical formation than lutein and zeaxanthin. The reason may be that the prolonged conjugated double bond chain of isoreineratene provides more reactive sites and electron for ∙OH radical. The isorenieratene molecule containing only carbon and hydrogen may also be easier to supply hydrogen atom to the quenching ∙OH than lutein and zeaxanthin. In addition, the aromatic structure might give isorenieratene higher stability and resistant ability against the relatively high dose UVB radiation.

### 2.2. Cytotoxic Effects of Isorenieratene and Macular Xanthophylls

**Figure 3:**
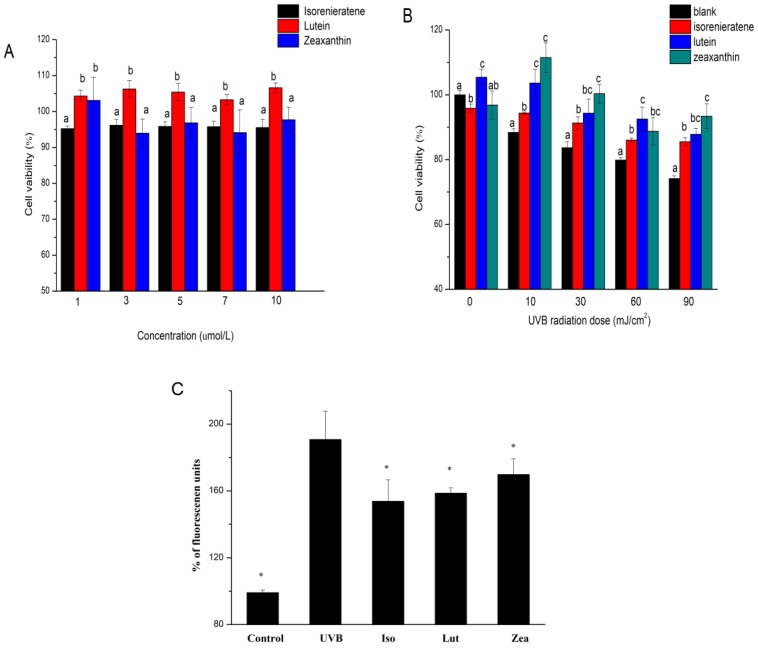
Cytotoxic effects of three different kinds of carotenoids; (**A**) ARPE-19 cell survive rate after UVB radiations with three different kinds of carotenoids; (**B**) cellular reactive oxygen species (ROS) generation in ARPE-19 cell after UVB radiations with three different kinds of carotenoids (**C**). Columns representing the same concentration or radiation dose with different letters (a, b or c) are significantly different (*p* < 0.05). * Significantly different from UVB group (*p* < 0.05).

The effects of three kinds of carotenoids on the ARPE-19 cell viability were shown in Figure 3A. As the concentrations were 1–10 μmol/L, neither isorenieratene nor the macular xanthophylls showed suppression effects on the viability of HFF-1 cells. These results indicated that isorenieratene could be used as a safe retinal protector the same with lutein and zeaxanthin in restricted does.

### 2.3. Isorenieratene Increases ARPE-19 Cell Survive Rate after UVB Radiation

The MTT assay was used to investigate the protective effects of the isorenieratene on ARPE-19 cells exposed to UVB irradiation. Compared to the non-irradiated cells, the cell viability of ARPE-19 after exposure to 10 mJ/cm^2^ UVB radiations was reduced to 88.4% (Figure 3B). When the UVB radiation was increased to 90 mJ/cm^2^, the cell viability decreased to 79.9%. All three carotenoids exhibited protective effects against UVB irradiation after 10, 30, 60 and 90 mJ/cm^2^ UVB radiations at concentrations of 5 μmol/L (Figure 3B). When the UVB dose increased, the cell viability with isorenieratene showed almost the same protective effects as macular xanthophylls.

### 2.4. ROS Production in ARPE-19 Cell

As shown in Figure 3C, after 30 min following UVB irradiation (90 mJ/cm^2^), the fluorescence intensities in cells greatly increased. Accordingly, enhanced ROS generations led to cell damage resulting in a significant decrease of the ARPE-19 cell viability (Figure 3B) after exposure to UVB irradiation. Pre-treatment of ARPE-19 cells with both isorenieratene and macular xanthophylls for 60 min showed a reduction in cellular ROS generation (Figure 3C). The cellular ROS levels decrease trend is analogous with the EPR measurement, which again proved the anti-oxidant effect of isorenieratene under UVB radiation. Consequently, the reduced oxidative stress condition could lead to an inhibitive effect in ARPE-19 cell death (Figure 3B). 

### 2.5. Cell Apoptosis Measurements

**Figure 4:**
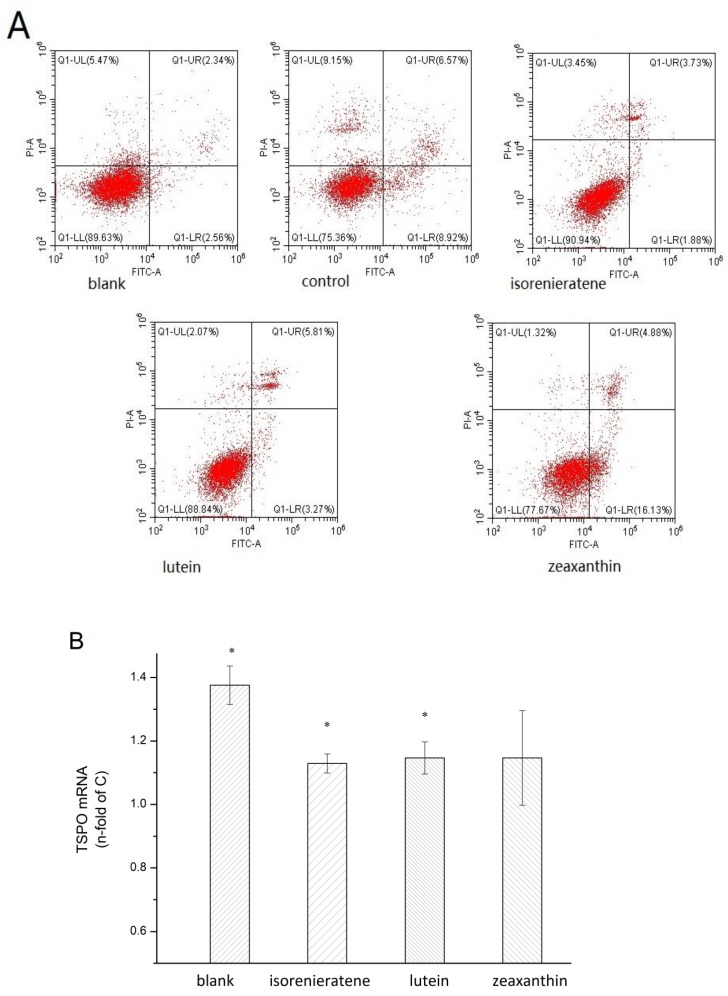
Flow cytometry, (**A**) real-time fluorescent quantitative PCR (**B**) analysis of ARPE-19 cell after UVB radiations (90 mJ/cm^2^) with three different kinds of carotenoids. * Significantly different from UVB control (n-fold = 1) group (*p* < 0.05).

To determine the protective effect of isorenieratene after UVB radiation in ARPE-19 cells, flow cytometry was performed to determine the percentage of cell apoptosis. After 24 h following UVB treatment (90 mJ/cm^2^), it was found that all three carotenoids showed the reducing trend on the percentage of apoptosis (Figure 4A). In addition, the results showed that isorenieratene possessed an excellent protective effect against UVB-induced ARPE-19 cell apoptosis. In addition, the proportion of viable cells with isorenieratene was slightly higher than the lutein group and much higher than the zeaxanthin group. These results suggested that isorenieratene can effectively drop UVB-mediated oxidative stress and lower apoptosis in ARPE-19 cells after 90 mJ/cm^2^ UVB radiation. 

### 2.6. Real-time Fluorescent Quantitative PCR and Western Blot Analysis

**Figure 5:**
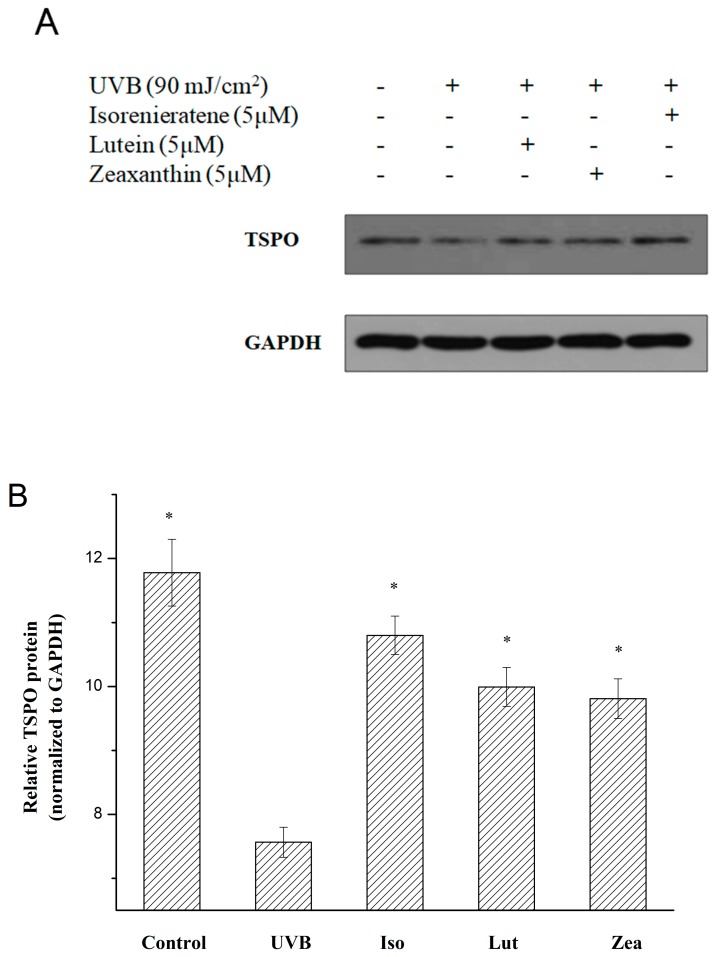
Western blot analysis of ARPE-19 cellafter UVB radiations (90 mJ/cm^2^) with three different kinds of carotenoids.(**A**) TSPO level; (**B**) relative protein expression of TSPO. * Significantly different from UVB group (*p* < 0.05).

Based on the previous reports, *tspo* knockout mice showed multiple morbific phenotypes and the TSPO protein is believed to play an essential role in the metabolic process including stress adaption, diminishing apoptosis, cholesterol transport etc. [21]. Thus, we performed a further study to explore the effects of UVB irradiation upon TSPO in ARPE-19 cells and the mRNA expression levels of TSPO were analyzed by the Real-Time fluorescent quantitative PCR. Results showed a lower expression level of TSPO mRNA after UVB irradiation (90 mJ/cm^2^) than the non-radiated group (blank). Additionally, as shown in Figure 4B, after pre-treatment with three different carotenoids, an obvious reduced effect of UVB-induced inhibition expression of TSPO was observed, which indicated that the internal protective mechanism might be related to the upregulating of the TSPO gene. Among three carotenoids, isorenieratene showed significantly increased TSPO mRNA level (1.13 ± 0.03 fold). To further explore the protective molecular mechanism against UVB-induced retinal damage, we investigated the TSPO protein level. In accordance with the Real-Time PCR results, the western analysis showed that the TSPO protein level was evidently decreased in ARPE-19 cells after UVB radiation (Figure 5). Meanwhile, western results also proved that with pre-treatment with isorenieratene, TSPO protein levels were significantly increased compared with the control group, and the isorenieratene group still showed the highest TSPO protein level. 

### 2.7. Molecular Docking

**Figure 6:**
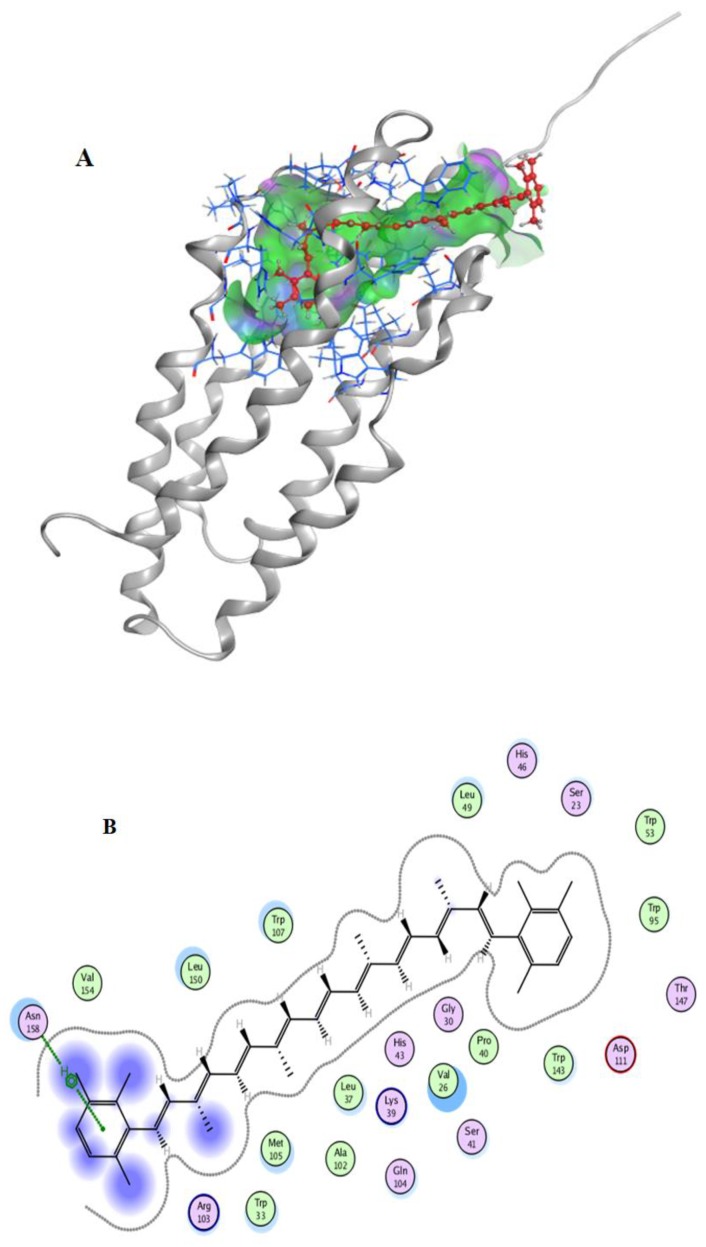
of isorenieratene (**A**) binding to hTSPO; molecular contacts between isorenieratene (**B**) and amino acids of hTSPO.

After the docking simulation, the structure of the most likely binding conformation and the 2D interaction diagram of hTSPO with ligands in site were captured. Figure 6A,B showed that isorenieratene could perfectly fit into the site. There are some hydrophobic interactions in the ligand interaction, and the residue Asn158 of hTSPO highly likely generated an arene-H interaction with isorenieratene. The S-score of this binding conformation is −8.5438. Figure S2A,B showed the interaction of lutein with hTSPO. There were also some hydrophobic interactions, and the residue Trp53 of hTSPO are intended to generate a hydrogen bond interaction with lutein. The S-score of this binding conformation is −5.2711. Figure S2C,D show the interaction of zeaxanthin with hTSPO. There were still some hydrophobic interactions in this interaction. The S-score of this binding conformation is −5.6872. In summary, these three kinds of carotenoids all might bind to the protein hTSPO in site. Especially, isorenieratene have a better binding conformation. 

Thus, we deduce that the fine binding activity of isorenieratene as a hTSPO ligand could be the reason of the high expression of *tspo* in ARPE-19 cells. The higher ROS inhibition rate and cell viability compared with the control group were in accord with the Biswas research [19].

According to Marcel’s research, the S1 lifetime and absorption spectra of isorenieratene revealed that the ϕ ring did no effective affects on the conjugation length [22]. It might be that the π-π interaction shortens the S1 state lifetime as well as for photo-protective quenching ability. Thus we deduce that the superior bioactivity of isorenieratene, including ROS reduction in ARPE-19 cells, quenching effects of ROS in multilamellar vesicles and fine binding conformation with TSPO protein, is mainly contributed by its two aromatic rings and their orientations in system. 

## 3. Materials and Methods

### 3.1. Materials and Reagents

1-palmitoyl-2-oleoylphosphatidylcholine (POPC, >99%) was purchased from Aladin Reagent Co., Ltd (Shanghai, China). Lutein (>90%), zeaxanthin (>90%), 5,5-dimethyl-1-pyrroline-N-oxide spine (DMPO, >97%), and 2,2,6,6-tetramethylpiperidine spine (TEMP, >97%) were purchased from Shanghai Source Biological Technology Co., Ltd (Shanghai, China). Isorenieratene (>90%) was purified from a carotenoid extract of *Rhodococcus* sp. B7740 using HSCCC [10]. Adult human retinal pigment epithelial cell (ARPE-19) was purchased from the China Center (Wuhan, China) for Type Culture Collection (CCTCC). 3-(4,5-dimethyl-2-thiazolyl)-2,5-diphenyl-2-H-tetrazolium bromide, Thiazolyl Blue Tetrazolium Bromide (MTT, >98%) and other chemicals were obtained from Sigma (Deisenhofen, Germany) unless stated otherwise.

### 3.2. Detection of Free Radicals by EPR Spectroscopy in Model Liposomes System

#### 3.2.1. Model Liposomes Preparation

Multilamellar vesicles were prepared according to the published article using the film deposition method [16]. Briefly, 1-palmitoyl-2-oleoylphosphatidylcholine (POPC) was firstly dissolved in chloroform. Then, POPC in chloroform was thoroughly dried under reduced pressure. In the end, to obtain the uniform suspension of lipids (2.5 mM), the PIPES buffer (pH = 7.0) with the fixed volume was added and vortexed. 

#### 3.2.2. Detection of Hydroxyl Radicals and Singlet Oxygen by EPR Spectroscopy

DMPO (0.1 g/mL in ultrapure water) and TEMP (0.1 g/mL in ultrapure water) were used to capture the hydroxyl radicals and singlet oxygen, respectively. Three kinds of carotenoids including lutein, zeaxanthin and isorenieratene (dissolved in DMSO) were diluted in multilamellar vesicles at the concentration of 50 μmol/L and 5 μmol/L, respectively. The free radical trap was mixed with the multilamellar vesicles with or without carotenoids in the same volume. Before EPR measurement, these mixtures were under a series dose of UVB radiation (90, 120, 150, 180, 240, 360, 600 mJ/cm^2^). The determination of hydroxyl radicals using EPR spectroscopy (Magnettech, Freiberg Instruments, Berlin, Germany) was performed as described by Wang et al. and Yin et al. with some modifications [23,24]. The parameters of EPR measurements were as follows: Centre field 338 mT; sweep width 10 mT; sweep time 60 s; microwave power 10 mW; modulation amplitude 200 mT.

### 3.3. Cell Culture

ARPE-19 cell lines were maintained in the growth medium DMEM/F12 supplemented with 10% fetal calf serum (FBS) and 1% penicillin/streptomycin (5000 U mL^−1^). Cells were cultured at 37 °C in a humidified atmosphere containing 5% CO^2^ [15].

### 3.4. Cytotoxicity Assay of Carotenoids

The cell viability was determined using the MTT assay [13,25,26]. One hundred μL of growth medium containing cells (2 × 10^5^ /mL) were plated in 96-well plates. After 24 h incubation, cells were treated with various concentrations (0, 1, 3, 5, 7, 10 μmol/L medium) of three kinds of carotenoids (lutein, zeaxanthin and isorenieratene dissolved in DMSO) in the FBS-free medium for 24 h and the DMSO remained at the nontoxic concentration (1‰, *v*/*v*) in each cell. After removing the supernate with carotenoid, each well was washed using 150 μL of PBS for three times. Further, 200 μL of serum free MTT media (0.5 mg/mL) was added to each well and the cells were incubated for 4 h at 37 °C. One-hundred-and-fifty μL of DMSO was added to each well after disposing the supernatant and the absorbance of each well was recorded at 490 nm using the Synergy HTX micro plate reader (BioTek, Winooski, VT, USA). 

### 3.5. ARPE-19 Cell Viability after Pre-treatment with Carotenoid and UVB Irradiation

After 24 h incubation (2 × 10^4^ per cell) in 96-well plates, ARPE-19 cells were treated with three different kinds of carotenoids (lutein, zeaxanthin and isorenieratene, 5 μmol/L) in the FBS-free medium for 1 h. Each well was washed using 150 μL of PBS for three times after removing the supernatant [13]. Further, 50 μL of PBS was added to each well followed by irradiation using UVB lamp (850 μw/cm^2^) with different doses (0, 10, 30, 60, 90 mJ/cm^2^). After irradiation, PBS was removed and 100 μL of FBS-free medium was added followed by 24 h incubation. The cell viability was measured using the MTT assay performed as described in the previous cytotoxicity assay. 

### 3.6. Flow Cytometry

Two mL of growth medium containing cells (2 × 10^5^ /mL) were plated in 6-well plates under the conditions mentioned above. The ARPE-19 cells were pre-treated with three different kinds of carotenoids (lutein, zeaxanthin and isorenieratene, 5 μmol/L) in the FBS-free medium for 1 h and irradiated with UVB (90 mJ/cm^2^). After 24 h incubation in the FBS-free medium, ARPE-19 cells was harvested and then treated according to the manufactory’s instructions using AnnexinV-FITC/PI Kit (Nanjing Keygen Biotech. Co., Ltd, China). The cells were analyzed by flow cytometry (Beckman Coulter, CytoFLEX, Brea, CA, USA) [27].

### 3.7. Reactive Oxygen Species (ROS) Generation Determination in ARPE 19 Cell

The intracellular ROS was measured according to the published paper with a few changes [13]. ARPE-19 cells were seeded in 96-well plates (dark) as described in the cytotoxicity measurement. The cells were treated with 5 μM lutein, zeaxanthin or isorenieratene for 1 h. After that, the cells were washed using 150 μL PBS for three times and incubated with the DCFH-DA probe (diluted in FBS free culture medium, 10 μmol/L) for 30 min. Then, each cell was washed using PBS for three times to remove the unabsorbed probe. The cells were then irradiated with UVB in different doses (0, 10, 30, 60, 90 mJ/cm^2^). After another 1 h of incubation, the fluorescence measurement was performed at an excitation wavelength of 485 nm and an emission wavelength of 530 nm using a plate reader (BioTek). 

### 3.8. Real-time PCR Analysis

Two mL of the growth medium containing cells (2 × 10^5^ /mL) were plated in 6-well plates under the conditions mentioned above. The pre-treatments with carotenoids and UVB exposure were the same with the cell viability assay. After the treated cells incubated in the FBS free-medium for 24 h, total RNA of ARPE-19 was extracted using Trizol reagent (GeneCopoeia, Rockville, MD, USA) according to the manufactory’s instructions [26]. cDNA was synthesized using a first-strand cDNA synthesis kit (GeneCopoeia, Rockville, MD, USA). The primer sequences were listed in Table 1. All samples were run in triplicates and the threshold cycle value (C_T_) was used to calculate the expression of mRNA. For each sample, ΔC_T_ sample value was calculated by analyzing the difference between C_T_ value of the target Homo TSPO gene and that of the Homo GAPDH reference gene. The relative expression levels were estimated by calculating the ΔΔC_T_ (ΔC_T_ sample-ΔC_T_ reference) and using the 2-^ΔΔC^^T^ method [28]. 

### 3.9. Western Blot Analysis

The pre-treatment and incubation of ARPE-19 cells were the same with the PCR analysis. Western blotting was performed as described previously [12]. The TSPO (Abcam plc, Cambridge, UK) was used as the primary antibody and GAPDH was used as an internal control. The intensity of bands was quantified using the Image Lab software (Bio-Rad, Berkeley, CA, USA). 

### 3.10. Docking Simulation

The docking simulations were performed using the Molecular Operating Environment (MOE), version2018 (Chemical Computing Group, Montreal, QC, Canada). In this study, due to unavailability of the PDB structure of human TSPO (hTSPO), its 3D structure was prepared using the Modeling server i-TASSER [29]. The site was predicted by Site Finderin MOE and typical residues including Cys19, Ser23, Phe25, Val26, His27, Gly30, Leu31, Trp33, Lys39, Pro40, Ser41, His43, Pro44, Pro45, His46, Leu49, Gly50, Trp53, Trp95, Ala102, Trp107, Val110, Asp111, Leu114, Trp143, Thr147, Leu150, and Val154 were chosen. According to previous research of Jaremko et al., the structure of the 18 kD translocator protein TSPO in mouse (mTSPO, PDB code: 2MGY) was presented, which share the similar binding residues of our predicted pocket in hTSPO [30]. 

Before docking, both the receptor and the ligands were prepared. The receptor (hTSPO) was performed, the LigX operation and the ligands were built and energy minimized by MOE. During docking, the following options should be set: For the first scoring function, choose “London dG” for Rescoring 1 and drop down its Retain option to 30; second, choose “Forcefield” for refinement; third, make sure the Rescoring 2 of the second scoring function is set to GBVI/WSA dG and the Retain is set to 30. After docking, the conformation with the low S-score was chosen as the potential binding conformation.

### 3.11. Statistical Analysis

Origin software 8.0 (Originlab Corporation, Northampton, MA, USA) was used to perform all statistical analyses and draw figures. All data were calculated using the one-way ANOVA of SPSS 17.0 (UNICOM Intelligence, Mission Hills, CA, USA) followed by the Tukey’s multiple-range test. Data were presented as means ± standard deviation (SD) and the statistical significance was defined as *p*< 0.05.

## 4. Conclusions

Over all, we have used both the multilamellar vesicles model and human retina cell model to analyze the potential role of isorenieratene against UVB-induced damage. The data above indicated that isorenieratene possesses splendid anti-oxidant ability and protective effect against UVB radiation compared with two macular xanthophylls. EPR results showed that isorenieratene could decrease the formation of both singlet oxygen and hydroxyl radicals in model liposomes system. MTT and flow cytometry results indicated the protective effects of isorenieratene on ARPE-19 in the UVB irradiation model. ROS, RT-PCR and WB analysis explained the molecular mechanisms of its protective effects including its antioxidant effect and interacting ability with TSPO. Additionally, molecular docking confirmed the possible binding property of isorenieratene with TSPO. Thus, we believe that isorenieratene, as one aromatic carotenoid may possess multiple functions, protecting retina and inhibiting UV-induced damage. However, this study measured the potential function of isorenieratene from certain perspectives. Thus, there still are obvious limitations and research niches, which make the research on isorenieratene remain urgent, especially its in vivo activity.

## Figures and Tables

**Table 1:** The primer sequences used in *tspo* cDNA analysis.

Name	Primer	Sequence	Size
Homo GAPDH	Forward	5′-TCAAGAAGGTGGTGAAGCAGG-3′	115 bp
	Reverse	5′-TCAAAGGTGGAGGAGTGGGT-3′	
Homo TSPO	Forward	5′-CATCTTCTTTGGTGCCCGAC-3′	163 bp
	Reverse	5′-GTTGAGTGTGGTCGTGAAGG-3′	

## Supplementary Materials

The following are available online at https://www.mdpi.com/1660-3397/17/6/316/s1, Figure S1: The singlet oxygen accumulations in model liposomes after 30 (A), 90 (B) mJ/cm^2^ of UVB irradiation with or without pretreatments of carotenoids, respectively, Figure S2: Interaction of lutein(A) and zeaxanthin(C) binding to hTSPO; molecular contacts between lutein(B) or zeaxanthin(D) and amino acids of hTSPO.
