# Supplementary material for: Potential TSPO Ligand and Photooxidation Quencher Isorenieratene from Arctic Ocean Rhodococcus sp. B7740
Source: Mar Drugs. 2019 May 29;17(6):316. doi: 10.3390/md17060316 (PMC6627809; doi:10.3390/md17060316)
Supplement: Supplementary file 1 [file marinedrugs-17-00316-s001.pdf]

# Figure S1

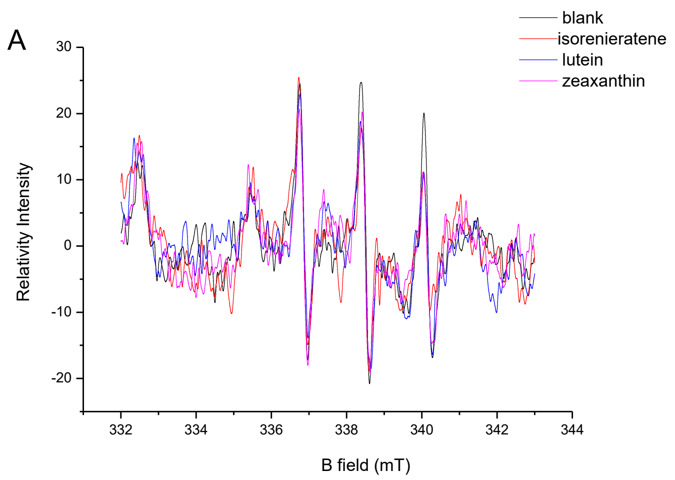


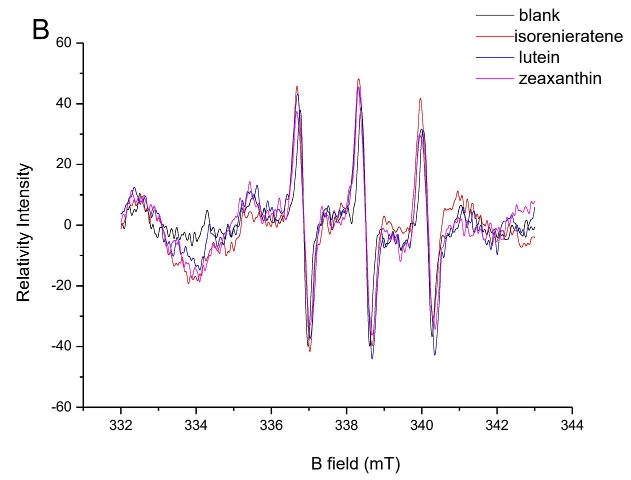


**Figure S1.** The singlet oxygen accumulations in model liposomes after 30 (A), 90 (B) mJ/cm$^2$ of UVB irradiation with or without pretreatments of carotenoids, respectively.

**Figure S2**

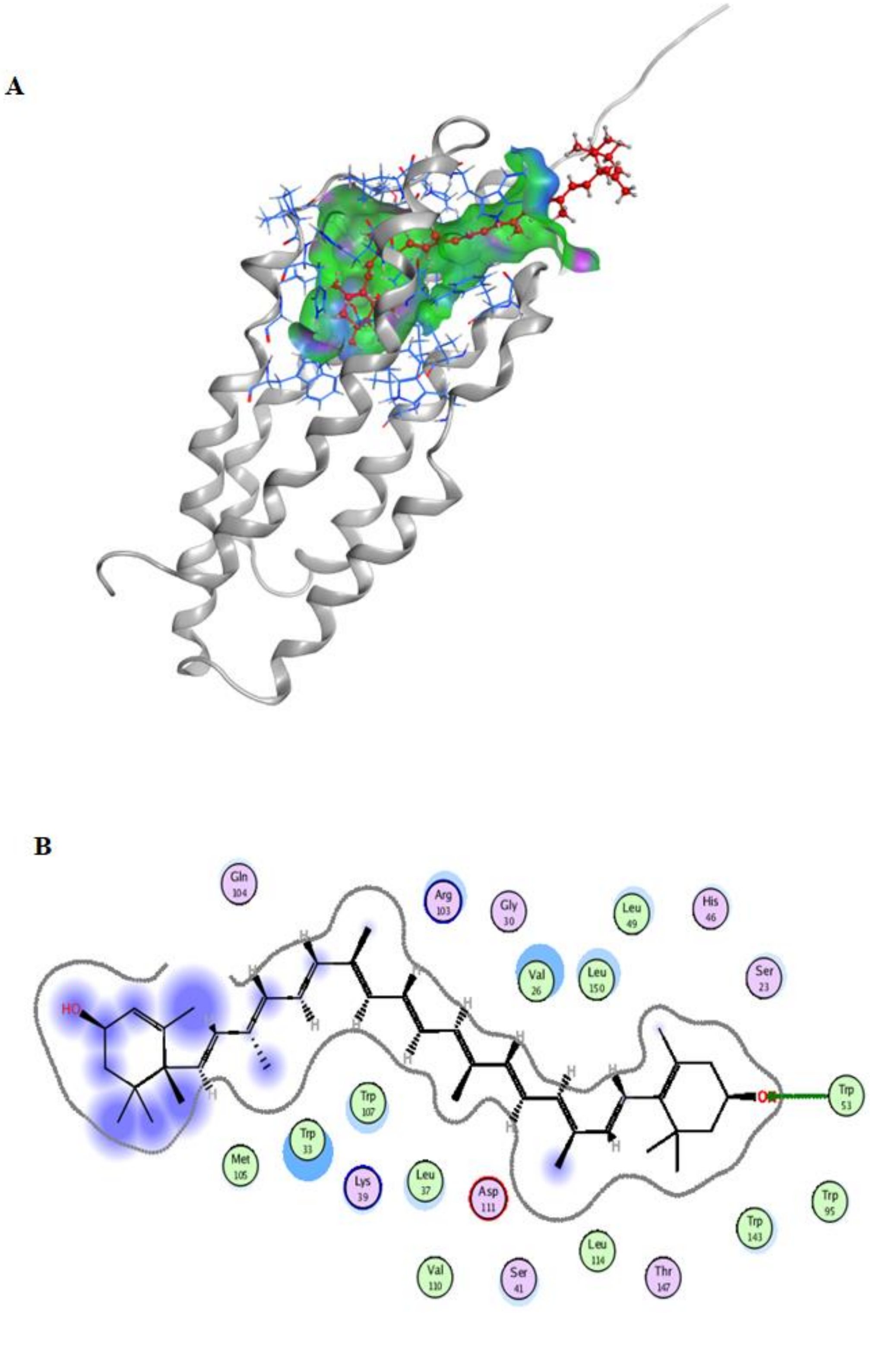
A
B
Gln 104
Arg 103
Gly 30
Leu 49
His 46
Val 26
Leu 150
Ser 23
HO
Trp 107
Trp 33
Met 105
Lys 39
Leu 37
Asp 111
Trp 53
Trp 95
Trp 143
Leu 114
Thr 147
Val 110
Ser 41

C

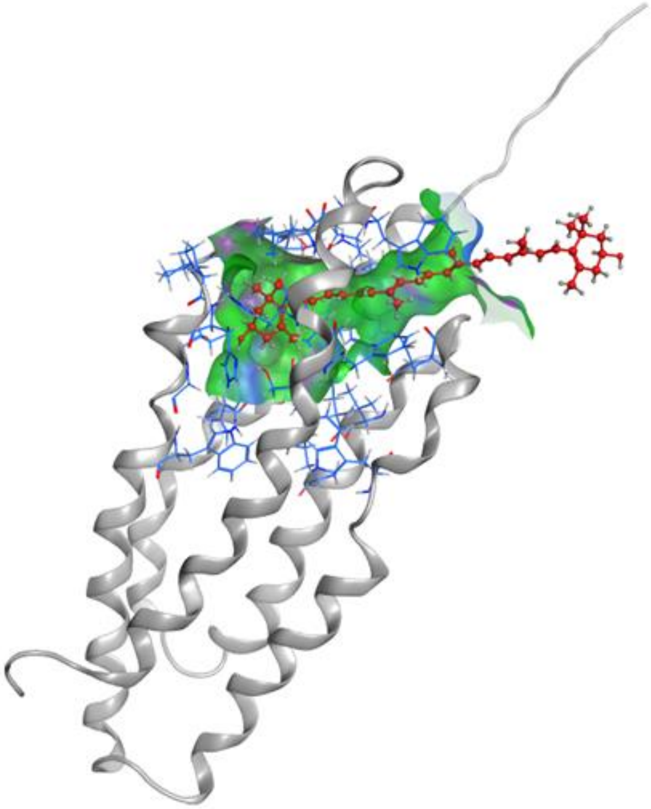

D

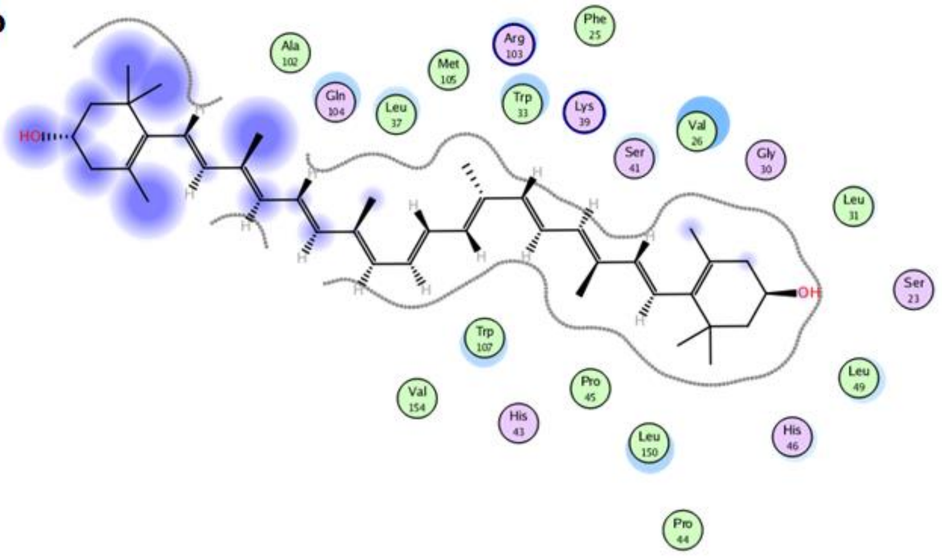


**Figure S2**. Interaction of lutein(A) and zeaxanthin(C) binding to hTSPO; Molecular contacts between lutein(B) or zeaxanthin(D) and amino acids of hTSPO.
